# Characterization of influenza virus sialic acid receptors in minor poultry species

**DOI:** 10.1186/1743-422X-7-365

**Published:** 2010-12-09

**Authors:** Brian Kimble, Gloria Ramirez Nieto, Daniel R Perez

**Affiliations:** 1 Department of Veterinary Medicine, University of Maryland College Park, and Virginia-Maryland Regional College of Veterinary Medicine, 8075 Greenmead Drive, College Park, MD 20742, USA; 2 Facultad de Medicina Veterinaria y Zootecnia, Universidad Nacional de Colombia, Carrera 30 No. 45-03, Edificio 561B, Bogota, Colombia

## Abstract

It is commonly accepted that avian influenza viruses (AIVs) bind to terminal α2,3 sialic acid (SA) residues whereas human influenza viruses bind to α2,6 SA residues. By a series of amino acid changes on the HA surface protein, AIVs can switch receptor specificity and recognize α2,6 SA positive cells, including human respiratory epithelial cells. Animal species, like pigs and Japanese quail, that contain both α2,3 and α2,6 SA become ideal environments for receptor switching. Here, we describe the SA patterns and distributions in 6 common minor domestic poultry species: Peking duck, Toulouse geese, Chinese ring-neck pheasant, white midget turkey, bobwhite quail, and pearl guinea fowl. Lectins specific to α2,3 and α2,6 SA *(Maakia amurensis *agglutinin and *Sambuca nigra *agglutinin, respectively) were used to detect SA by an alkaline phosphotase-based method and a fluorescent-based method. Differences in SA moieties and their ability to bind influenza viruses were visualized by fluorescent labeling of 4 different H3N2 influenza viruses known to be specific for one receptor or the other. The geese and ducks showed α2,3 SA throughout the respiratory tract and marginal α2,6 SA only in the colon. The four other avian species showed both α2,3 and α2,6 SA in the respiratory tract and the intestines. Furthermore, the turkey respiratory tract showed a positive correlation between age and α2,6 SA levels. The fact that these birds have both avian and human flu receptors, combined with their common presence in backyard farms and live bird markets worldwide, mark them as potential mixing bowl species and necessitates improved surveillance and additional research about the role of these birds in influenza host switching.

## Introduction

Waterfowl act as the natural reservoir of influenza A viruses. Virus isolates from these birds show high binding preference towards glycans that terminate in sialic acids linked to galactose in an α2,3 conformation (α2,3 SA), the same receptor that dominates the duck intestinal and respiratory tracts [1,2]. These isolates typically show low infectivity in humans due in part to the prevalence in the respiratory tract of glycans terminating in sialic acid (α2,6) galactose (α2,6 SA) [3,4]. However, stable, species specific, viral lineages have jumped from the natural reservoir to wild non-aquatic birds, domestic poultry, and many mammalian species, most notably swine and humans.

In order for an avian virus to infect a human, several changes must occur in the virus, most notably in the HA protein. This can happen in one of two ways: the build up of specific mutations (genetic/antigenic drift) or the recombination with a second virus with a suitable HA gene (genetic/antigenic shift). Both of these processes are facilitated by infection in a 'mixing bowl' species, a host that can accommodate both types of receptors. For example, swine express both sialic acid moieties and allowed it to play a critical role in the current H1N1 pandemic [2,5].

The emergence of highly pathogenic avian influenza (HPAI) in people who have direct contact with poultry underscore the role poultry play in the transmission of influenza into humans, yet very little is known about the distribution of sialic acid receptors in most poultry species [6,7]. Thus, little is known of the potential of poultry species to act as mixing bowls. Previous studies have shown that mallard and Peking ducks display predominately α2,3 SA in both the intestinal tract and the respiratory tract [8-10]. White leghorn chicken and, particularly, Japanese quail show more α2,6 SA expression in the respiratory tract [9,11].

Typically, plant lectins that specifically bind to terminal SA are used to identify the distribution of SAs in tissues via lectin histochemistry. *M. amurensis *agglutinin (MAA) binds most predominantly to any glycan terminating in α2,3 SA while *S. nigra *agglutinin binds to terminal α2,6 SA [12,13]. Here we use two methods of lectin staining to describe the distribution of α2,3 SA and α2,6 SA in six poultry species: Peking duck, Toulouse goose, Chinese ring-neck pheasants, white midget turkey, bobwhite quail, and pearl guinea fowl. The first method is based on digoxigenin-linked lectins and HRP (horseradish peroxidase)-linked anti-digoxigenin antibodies that interact with a substrate to precipitate a marker visible by light microscopy. The second is based on fluorescently-labeled lectins that are visible under a fluorescent microscope.

These methods, however, do not directly measure a tissues capacity to bind influenza virus as there are many other variables that determine binding ability. Specific amino acid sequence and glycosylation in and near the receptor binding site of HA can shift binding specificity from α2,3 SA to α2,6 SA and vice versa. Additionally, these changes can shrink or expand the pool of specific glycans terminating in α2,3 SA or α2,6 SA that HA can bind [14,15]. Various modifications to the receptors can also change binding specificity [16,17]. To assuage these issues, we also used a virus-binding histochemistry technique to directly measure the virus binding patterns as they correlated to the SA distribution.

## Animal tissues

One day-old Peking ducks, Toulouse geese, Chinese ring-neck pheasants, white midget turkeys, bob white quail, and pearl guinea fowl were received from McMurray Hatchery (Webster City, IA). Animals were maintained in ABSL2 conditions in the Department of Veterinary Medicine for 4 weeks. In the case of ducks and geese, one animal was sacrificed for tissue collection at the age of 1, 2 and 4 weeks of age. For all other birds 2 animals were sacrificed for tissue collection at 1, 2, and 4 weeks of age. Japanese quail were hatched at the Department of Veterinary Medicine and maintained in ABSL2 conditions for 4 weeks. Two animals were sacrificed for tissue collection. The Institutional Animal Care and Use Committee of the University of Maryland, College Park, approved all animal studies. Animal studies adhere strictly to the US Animal Welfare Act (AWA) laws and regulations.

## Viruses

A/duck/Hong Kong/375/1975 (H3N2) and A/turkey/Ohio/313053/2004 (H3N2) were kindly provided by Robert Webster, St Judes Children's Research Hospital, Memphis, TN and Yehia Saif, Ohio State University, Wooster, OH, respectively. These viruses were grown in 10 day old embrionated chicken eggs and stocks prepared and maintained at -70°C until use. A/Memphis/31/1998 (H3N2) was propogated in MDCK cells, stocks prepared and maintained at -70°C until use.

## Tissue preparation and sectioning

Trachea, lung, middle, and lower intestine were collected from each animal and rinsed in PBS for 5 minutes. Appropriate sized samples were wrapped in aluminum foil and frozen on dry ice. Samples were embedded in OCT and cut into 5 μm thick sections by Histoserv (Germantown, MD).

## Digoxigenin sialic acid (SA) detection method

Slides containing sections of tissue were rinsed for 1 h at room temperature in tap water before being fixed for 15 minutes in cold acetone followed by a 15 minute incubation in 2% H_2_O_2 _in methanol. Slides were rinsed 3 times for 5 minutes in tris-buffered saline (TBS) buffer and blocked over night at 4°C in 1% BSA (Sigma, Lenexa, KS) in TBS. Tissue was stained using DIG glycan differentiation kit (Roche, Mannheim, Germany). Briefly, slides were incubated for 1 hour at room temperature in digoxigenin (DIG)-labeled *M. amurensis *agglutinin (MAA, specific for α2,3SA) or DIG-labled *S. nigra *agglutinin (SNA, specific for α2,6 SA) in TBS. Following 3 rinses in TBS, slides were then incubated for 1 hour in peroxidase labeled anti-DIG fragments at room temperature. Three more washes in TBS were followed by 10 minute incubation in aminoethylcarbazole (AEC) (DAKO, Glostrup, Denmark) and counterstained in hematoxylin for 30 minutes. Cover slips were mounted using aqueous mounting media and tissues were observed under 400× magnification.

## Fluorescent sialic acid detection method

Slides were fixed and blocked similarly as described for the DIG-based method. Tissues were stained by incubating in FITC-labeled SNA (EY Laboratories, San Mateo, CA) and TRITC-labeled MAA or FITC-labeled MAA and TRITC-labeled SNA for 1 hour at room temperature. Following 3 rinses in TBS, slides were stained for 5 minutes in DAPI (4',6-Diamidino-2-phenylindole, dihydrocholride from Thermo Scientific Rockford, IL). Cover slips were mounted over the tissue using fluorescent mounting media (KPL, Gaithersburg, MD) and imaged at 400× or 630× magnification.

## Virus binding assay

Allantoic fluid or tissue culture supernatant was harvested and concentrated using the Centricon Plus-70 system from Millipore (Billerica, MA). Tissue was fixed and blocked as described in Digoxigenin sialic acid detection section. Approximately 600 HAU of virus was mixed 1:1 with 1% BSA in PBS and incubated on the tissue at 37°C for 2 hours. The virus was fixed after rinsing with 50/50 acetone/methanol for 15 min. at -20°C. The tissue was then incubated for 1 hour at room temperature with a monoclonal antibody specific to NP. Following three washes in phosphate buffered solution (PBS), the tissue was incubated in FITC-labeled anti-mouse antibody for one hour at room temperature in the dark. The tissue was then stained with DAPI and visualized with a fluorescent microscope at 400×.

## Results and Discussion

### Waterfowl and land land based poultry species differ in sialic acid distribution in various tissues

Lectin-based staining assays were used to determine the variations in sialic acid form and tissue distribution in various poultry species. Trachea, lung, and large intestine from 6 minor poultry species were used to determine the distribution of SA receptors. Ducks were included as a control as it has previously been reported that they show predominantly α2,3 SA in the trachea with increasing α2,6 on epithelial lining farther along the respiratory tract and only minimal α2,6 in the large intestine [10]. All other species were chosen for their presence in live poultry markets across the world.

The results indicate that there is a distinct difference between waterfowl (duck and goose) and land-based poultry (pheasant, turkey, bobwhite quail, and guinea fowl) (Table 1) in terms of presence and distribution of SA receptors, particularly α2,6. There were also age-based differences observed, particularly in turkeys (Table 1).

**Table 1:** Relative expression of sialic acid in avian tissues.

Species	Age (Week)	Trachea		Lung		Large intestine	
		
		2,3	2,6	2,3	2,6	2,3	2,6
Duck	1	**+**	**-**	**+**	**+**	**+**	**-**
	2	**+**	**-**	**++**	**+**	**++**	**+**
	4	**+**	**-**	**++**	**+**	**++**	**+**
Goose	1	**+**	**-**	**+**	**+**	**+**	**-**
	2	**+**	**-**	**+**	**+**	**++**	**+**
	4	**+**	**-**	**+**	**-**	**+**	**+**
Pheasant	1	**++**	**+**	**+**	**+**	**+**	**+**
	2	**++**	**+**	**+**	**+**	**+**	**+**
	4	**++**	**+**	**+**	**+**	**+**	**+**
Turkey	1	**++**	**+**	**+**	**+**	**+**	**-**
	2	**++**	**+**	**+**	**+**	**+**	**-**
	4	**++**	**++**	**++**	**++**	**+**	**-**
Guinea fowl	1	**+**	**+**	**+**	**+**	**+**	**-**
	2	**+**	**+**	**+**	**+**	**+**	**-**
	4	**+**	**+**	**+**	**+**	**+**	**-**
Quail	1	**+**	**+**	**+**	**+**	**+**	**-**
	2	**+**	**+**	**+**	**+**	**+**	**-**
	4	**+**	**+**	**+**	**+**	**+**	**-**

- no expression, + minimal expression, ++ moderate-high expression

**Figure 1:**
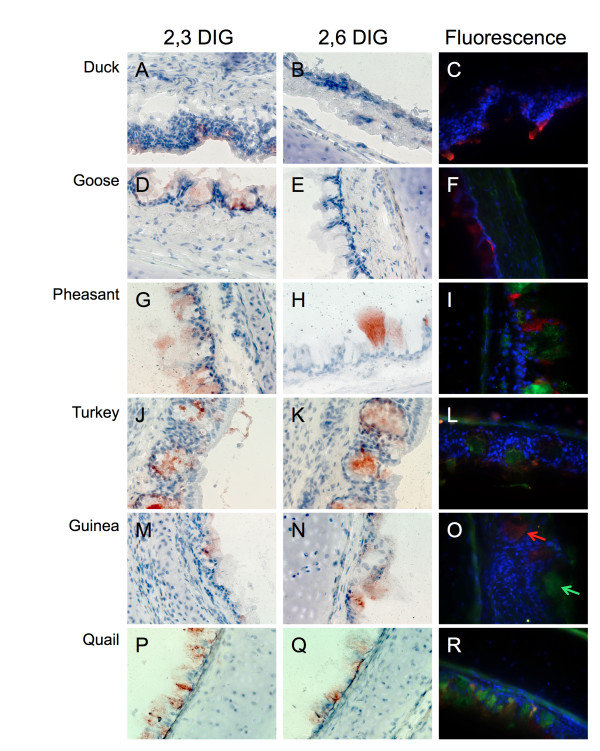
**Sialic acid distribution in avian trachea**. Representative sections of trachea from 4 week old duck (A, B, C), goose (D, E, F), pheasant (G, H, I), turkey (J, K, L), quail (M, N, O), and guinea fowl (P, Q, R) stained with either DIG labeled MAA (α2,3 specific, first column), DIG labeled SNA (α2,6 specific, second column) or FITC SNA (green α2,6) and TRITC MAA (red α2,3). Duck and goose trachea show only α2,3 SA while all other birds display both α2,3 and α2,6 SA.

**Figure 2:**
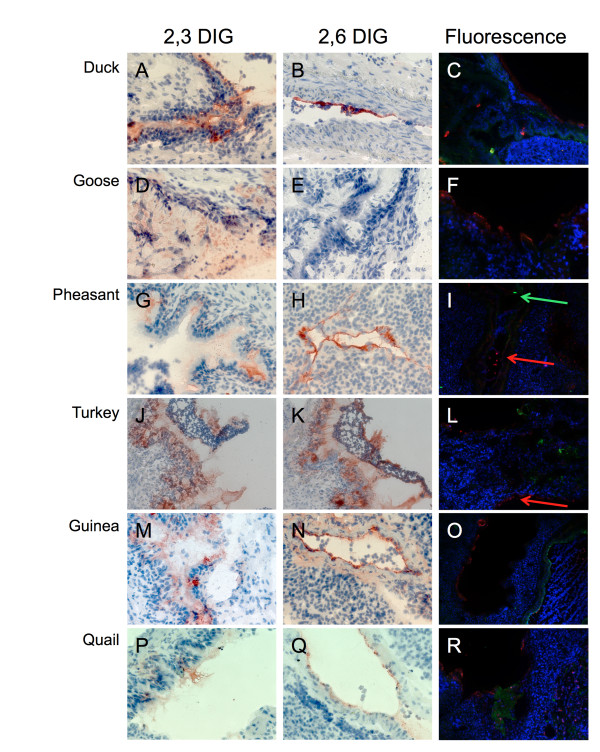
**Sialic acid distribution in avian lung**. Representative sections of lung from 4 week old duck (A, B, C), goose (D, E, F), pheasant (G, H, I), turkey (J, K, L), quail (M, N, O), and guinea fowl (P, Q, R) stained with either DIG labeled MAA (α2,3 specific, first column), DIG labeled SNA (α2,6 specific, second column) or FITC SNA (green α2,6) and TRITC MAA (red α2,3). Goose lung shows only α2,3 SA while all other birds display both α2,3 and α2,6 SA.

In the trachea, the ducks showed moderate to high levels of α2,3 SA (Table 1 and Figure 1A, B, C), consistent with previous reports [10,18]. There was no expression of α2,6 SA, consistent with one report [10], but not the other [18]. The geese trachea also showed an abundance of α2,3 SA and absence of α2,6 SA at any age (Table 1 and Figure 1D, E, F). On the contrary, the four land-based species showed both forms of sialic acid at all ages tested with positive staining of mucin-producing cells lining the lumen of the trachea (Table 1 and Figure 1G-R). Farther down the respiratory tract, the lungs (Figure 2) tested positive for both SA forms in all birds of all ages with the only exception being in the goose. Staining was present on cells lining the lumen of the lungs. Strong positive staining for both types of SA receptors was observed in the lungs of turkeys (Figure 2J, K), consistent with the observation of influenza outbreaks in turkeys caused by swine influenza viruses with human-like receptor specificity. The lungs of guinea fowl showed also significant staining for both SA receptors, which is consistent with the circulation in these birds of H9N2 viruses with human-like receptor specificity. At 4 weeks of age, no α2,6 SA was detected in the goose's lung (Figure 2E, F). However, both α2,3 SA and α2,6 SA were seen in the lung samples from geese at weeks 1 and 2 (not shown).

**Figure 3:**
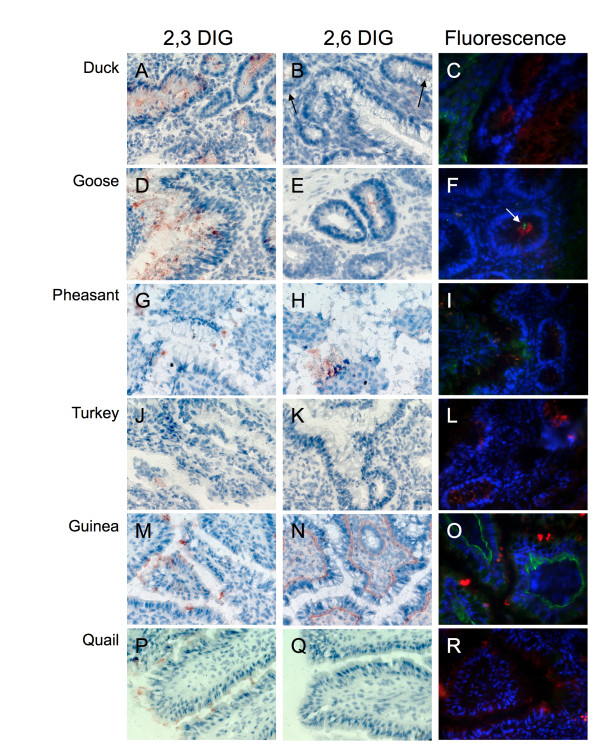
**Sialic acid distribution in avian large intestine**. Representative sections of large intestine 4 week old from duck (A, B, C), goose (D, E, F), pheasant (G, H, I), turkey (J, K, L), quail (M, N, O), and guinea fowl (P, Q, R) stained with either DIG labeled MAA (α2,3 specific, first column), DIG labeled SNA (α2,6 specific, second column) or FITC SNA (green α2,6) and TRITC MAA (red α2,3). Duck, goose, and pheasant large intestine show both α2,3 SA and α2,6SA while the other species show only α2,3SA. Arrows highlight positive reactions.

Testing of the large intestine once again showed a divide between the species. All six species tested positive for α2,3 SA in the large intestine in cells facing the lumen (Figure 3). However, duck, goose, and pheasant large intestine also showed minimal positive results for α2,6 SA (Figure 3B, E and 3H) while turkey, guinea fowl and quail tested negative (Figure 3K, N and 3Q; please note that significant α2,6 SA staining was observed on the basolateral side - opposite to the intestinal lumen - of epithelial cells in guinea fowl.)

The birds can be divided into three groups based on the distribution of sialic acids in the tissues examined. The waterfowl, the natural host of avian influenza viruses, show predominantly α2,3 SA in their tissues. α2,6 SA is only seen in the lower respiratory tract and minimally in the large intestine. The land-based birds also express α2,3 SA in all the tissues tested, however, they also express significant levels of α2,6 SA in the upper respiratory tract. This could help explain why these birds are susceptible to AIVs resulting in the emergence of strains with altered receptor specificity, including with human-like receptor binding [19]. This also underscores the potential role of these birds in influenza virus reassortment. Finally, the pheasants showed α2,6 SA in the trachea similar to the other land birds, but also showed α2,6 SA in the large intestine like the aquatic birds. This could make the pheasant more likely than other species to facilitate viral reassortment or to act as a "mixing bowl" species.

### Age dependent variations in α2,6 SA expression

**Figure 4:**
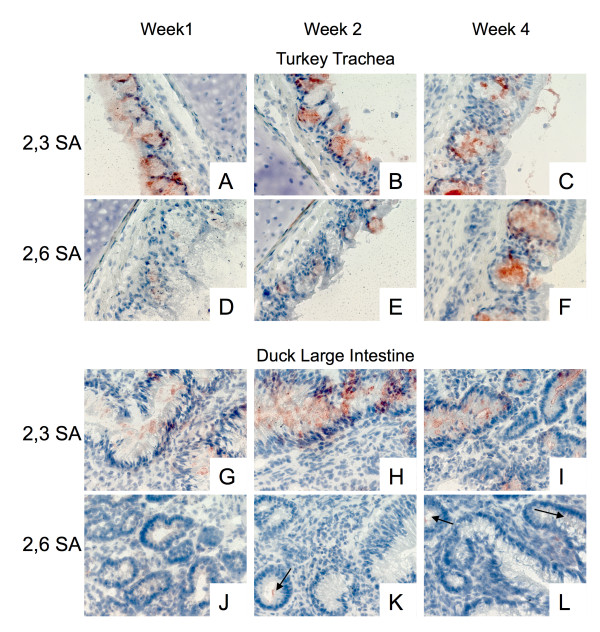
**Effects of age on sialic acid distribution**. Sections from 1, 2, and 4 week old turkeys trachea (A-F) and 1, 2, and 4 week old duck large intestine (G-L) were stained with either DIG labeled MAA (α2,3 specific, A-C and G-I) or DIG labeled SNA (α2,6 specific, D-F and J-L). Little to no variation was seen in the staining of α2,3 SA in the turkey trachea or duck large intestine across the age range. However, both species show an increase in α2,6SA as the birds age. Arrows highlight positive reactions.

While performing the experiments described above a trend was noticed in three species. The ducks and geese showed an increasing expression of α2,6 SA in the large intestine as they aged. Similarly, an increase in α2,6 SA detection was seen in the trachea of turkeys as they aged. The age dependence in turkeys was later reported by Pillai and Lee [18], however, they did not see any increase in α2,6 in Pekin ducks. There was no detection of α2,6 SA in the large intestine of ducks and geese at week 1 (Figure 4J for duck, not shown for geese). However, by week 2 there was a very low level positive reaction and at week 4 this reaction was slightly increased (Figure 4K and 4L arrows). Expression levels of α2,3SA remained relatively constant (Figure 4G-I) at all three time points.

In the turkey trachea this change in expression was even more pronounced. At week 1 (Figure 4D) only minimal α2,6 SA was detected. A week later (Figure 4E) there was a moderate positive response. By week 4 (Figure 4F) there was high level of expression. Again, there was no change in expression α2,3 SA at all time points (Figure 4A-C). No major age-related changes were observed in the other avian species tested for either 2,3 or 2,6 SA expression. This changing receptor pattern could have effects for live attenuated vaccines against viruses with a α2,6 binding preference in young turkeys and *in ovo *inoculations.

### Lectin binding patterns are not indicative of virus binding patterns

Glycan micro arrays have shown that not all α2,3 SA or α2,6 SA bind to influenza HA proteins equally well [15]. One glycan terminating in α2,3 SA might not bind HA while another may bind exceedingly well [15]. Unfortunately, both will show a positive reaction to the lectin-binding assays. Thus, determining the influenza virus-binding profile in tissues of different animal species is a condition *sine qua non *to better understand the role of these receptors.

Three H3N2 influenza viruses were selected to determine the correlation between lectin binding and virus binding using 3 prototypic H3N2 viruses to ensure differences were due to receptor specificity and not differences between subtypes. To determine the binding affinity of each virus, hemaglutinin agglutination assays were performed for each virus. According to previous reports, horse red blood cells (RBCs) express solely α2,3 SA on their surface while pig RBCs express predominantly α2,6 SA[20]. By comparing HA titers determined with each blood type, a binding preference can be ascertained. A/Dk/HK/7/75 (A/Dk) is a typical AIV duck isolate that bound horse RBCs twice as readily as pig RBCs, indicating a strong α2,3SA preference (Table 2). A/Tk/OH/313053/04 (A/Tk) was isolated from a turkey and bound pig RBCs slightly higher than horse RBCs, indicating a slight preference for α2,6SA (Table 2). A/Memphis/31/98 (A/Mem) is a human origin virus that shows no α2,3SA binding[21]. Accordingly, A/Mem only showed HA titer with the pig RBCs (Table 2). Using these three viruses we were able to determine the accuracy and resolution of the lectin binding results.

**Table 2:** Hemaglutinin binding affinity of H3N2 viruses.

	**Horse Red Blood Cells**		**Pig Red Blood Cells**	
	
	HA titer*	**StDv**	HA titer*	**StDv**
		
A/DK	64	0	32	0
A/TK	3	± 1.15	7	± 2
A/Memphis	0	0	20	± 8

*Average of 4 assays

**Figure 5:**
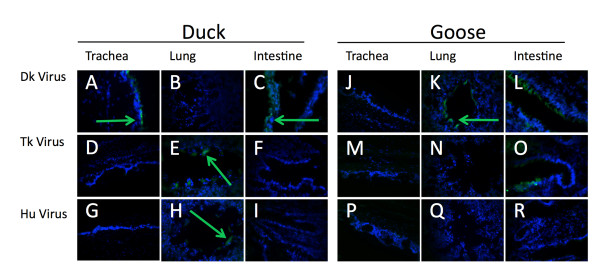
**Viruses binding to tissues correlates to sialic acid distribution in domestic ducks and geese**. Sections from 4 week old Peking duck (A-I) and Toulouse goose(J-R) tissues were exposed to A/DK/HK/7/75 (A-C, J-L), A/TK/OH/313053/04 (D-F, M-O), or A/Memphis/31/98 (G-I, P-R). Virus presence (green) was detected by αNP monoclonal antibodies and FITC linked α-mouse antibodies. Cells nuclei were stained with DAPI (blue).

**Figure 6:**
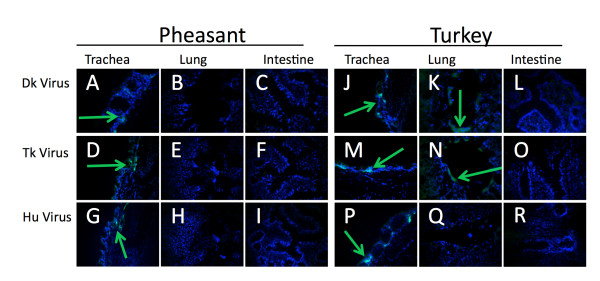
**Viruses binding to tissues correlates to sialic acid distribution in domestic turkeys and pheasant**. Sections from 4 week old white midget turkey (A-I) and Chinese ringneck pheasants(J-R) tissues were exposed to A/DK/HK/7/75 (A-C, J-L), A/TK/OH/313053/04 (D-F, M-O), or A/Memphis/31/98 (G-I, P-R). Virus presence (green) was detected by αNP monoclonal antibodies and FITC linked α-mouse antibodies. Cells nuclei were stained with DAPI (blue).

**Figure 7:**
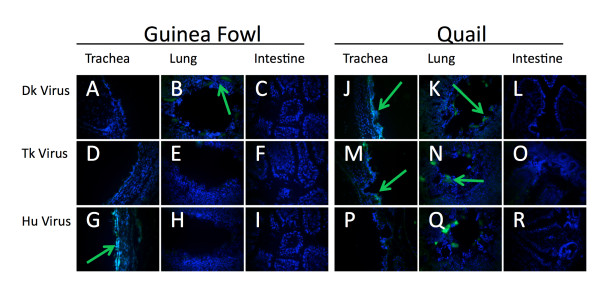
**Viruses binding to tissues correlates to sialic acid distribution in domestic quail and guinea fowl**. Sections from 4 week old bobwhite quail (A-I) and pearl guinea fowl (J-R) tissues were exposed to A/DK/HK/7/75 (A-C, J-L), A/TK/OH/313053/04 (D-F, M-O), or A/Memphis/31/98 (G-I, P-R). Virus presence (green) was detected by αNP monoclonal antibodies and FITC linked α-mouse antibodies. Cells nuclei were stained with DAPI (blue).

The trachea of the duck and geese showed no α2,6 SA. The virus-binding assay showed no binding to the A/Mem or the A/Tk viruses (Figure 5A, D, J and 5M). Additionally, there was minimal binding of A/Dk to the duck trachea (Figure 5G) and no virus binding of the A/Dk to the goose trachea despite ample expression of α2,3SA (Figure 5P). This is not unexpected as the typical route of infection in waterfowl is through the cloacae. In contrast, pheasant and turkey trachea exhibited the ability to bind all three viruses (Figure 6A, D, G, J, M and 6P). Based on fluorescent intensity and distribution of the fluorescent signal, in the pheasant the A/Dk virus showed the lowest levels of binding while the turkey showed equal binding between the three viruses. The quail trachea showed low binding with A/Dk and A/Tk, and no binding of the human A/Mem virus (Figure 7J, M, and 7P). The guinea fowl, on the other hand showed low levels of binding with A/Mem but no binding with A/Dk or A/Tk (Figure 7A, D and 7G).

To visualize the virus binding in the lungs, we imaged transversal sections of the parabronchi to minimize variations from section to section and from species to species. Whenever virus was seen in these sections, it was seen binding to the smooth atrial muscles lining the parabronchi regardless of bird species or virus. The lungs of ducks showed moderate binding of A/Tk and A/Mem but no binding to A/Dk (Figure 5B, E and 5H). The goose lung however showed binding with A/Dk (Figure 5K) but no binding with the other two viruses (Figure 5N and 5Q). Pheasants showed no binding of any virus in the parabronchi (Figure 6B, E and 6H). Turkey showed low to moderate binding of A/Dk and A/Tk but no binding of A/Mem (Figure 6K, N and 6Q) while the guinea fowl had A/Dk binding but neither of the other two viruses (Figure 7B, E and 7H). Finally the quail were the only species to show binding of all three viruses in the lungs (Figure 7K, N and 7Q).

Despite the fact that all birds expressed α2,3 SA in the intestines, only the ducks and the geese showed any ability to bind A/Dk in the intestines. The four land based poultry species showed no binding despite showing expression of α2,3SA. The duck, goose and pheasant intestines also showed minor α2,6 SA expression. However, only A/Tk was able to bind and only in the intestines of the geese (Figure 5L). These results highlight the complexities associated with understanding the host range of influenza viruses. Although many studies, including ours, have looked at the expression of SA receptors in tissues of several animal species, these receptors are not necessarily capable of binding influenza viruses (at least not under the conditions tested in this report). More studies are needed to better ascertain to which extent different animal species are likely hosts of influenza viruses and which minimal changes in receptor binding are needed to establish productive infections in these hosts.

## Competing interests

The authors declare that they have no competing interests.

## Authors' contributions

BK carried out the animal care, tissue staining, virus binding assays and drafted the manuscript. GRN carried out the animal care and participated in the study design. DRP conceived of the study, and participated in its design and coordination. All authors read and approved the final manuscript.
